# Functional dissection of synaptic circuits: *in vivo* patch-clamp recording in neuroscience

**DOI:** 10.3389/fncir.2015.00023

**Published:** 2015-05-22

**Authors:** Can Tao, Guangwei Zhang, Ying Xiong, Yi Zhou

**Affiliations:** Department of Neurobiology, Chongqing Key Laboratory of Neurobiology, Third Military Medical UniversityChongqing, China

**Keywords:** *in vivo*, patch-clamp, neural circuits, synaptic contribution, sensory cortex

## Abstract

Neuronal activity is dominated by synaptic inputs from excitatory or inhibitory neural circuits. With the development of *in vivo* patch-clamp recording, especially *in vivo* voltage-clamp recording, researchers can not only directly measure neuronal activity, such as spiking responses or membrane potential dynamics, but also quantify synaptic inputs from excitatory and inhibitory circuits in living animals. This approach enables researchers to directly unravel different synaptic components and to understand their underlying roles in particular brain functions. Combining *in vivo* patch-clamp recording with other techniques, such as two-photon imaging or optogenetics, can provide even clearer functional dissection of the synaptic contributions of different neurons or nuclei. Here, we summarized current applications and recent research progress using the *in vivo* patch-clamp recording method and focused on its role in the functional dissection of different synaptic inputs. The key factors of a successful *in vivo* patch-clamp experiment and possible solutions based on references and our experiences were also discussed.

## Introduction

The patch-clamp recording technique was originally developed to study currents from single ion channels in cell membranes in the 1970s. Over the last several decades, neuroscientists have successfully applied this technique to study current and potential changes in isolated cells, cultured cells and brain slice preparations, which has increased our knowledge of neuronal activity and circuit functions (Hamill et al., 1981). In recent years, the studies of the function and underlying circuit mechanisms of intact brain networks, especially in living animals, are drawing more and more attention because this is a critical step to fully understand the neuronal network. While novel experimental methods are rapidly revolutionizing the field, the *in vivo* patch-clamp recording method could still be the best available choice to directly measure synaptic contributions. Different types of neuronal activity, such as spiking responses, membrane potential dynamics and synaptic inputs from excitatory and inhibitory circuits, can be recorded from the same neuron using *in vivo* patch-clamp. By comparing the synaptic input and spiking output, the synaptic contributions to certain functions can be dissected and quantified functionally.

*In vivo* patch-clamp has been successfully applied in different regions of different species, including mouse (Ma et al., 2010; Nagtegaal and Borst, 2010), rat (Jacob et al., 2007; London et al., 2010), cat (Yu and Ferster, 2013), tadpoles (Zhang et al., 2000), Drosophila (Liu and Wilson, 2013; Murthy and Turner, 2013), *C. elegans* (Ramot et al., 2008), leopard frog (Rose et al., 2013) and zebrafish (Drapeau et al., 1999; Wei et al., 2012). In rat and mice, *in vivo* patch-clamp has been widely used to study circuitry functions and mechanisms in sensory cortices, including barrel cortex (London et al., 2010), auditory cortex (Li et al., 2013; Zhou et al., 2014), and visual cortex (Li et al., 2014b) as well as in the olfactory bulb (Poo and Isaacson, 2011), thalamus (Brecht and Sakmann, 2002; Margrie et al., 2002), hippocampus (Atallah and Scanziani, 2009; Grienberger et al., 2014), inferior colliculus (Nagtegaal and Borst, 2010; Kuo and Wu, 2012), spinal cord (Sonohata et al., 2004) and dorsal root ganglion (Ma et al., 2010). In *Drosophila*, *in vivo* patch-clamp has been used to study sensory systems, such as the medulla cortex (Behnia et al., 2014) and antennal lobe (Liu and Wilson, 2013). There are also applications of this method in zebrafish and *C. elegans* used to study the properties of neuronal and circuit function (Drapeau et al., 1999; Ramot et al., 2008). In *C. elegans*, it has been shown that neurons do not use action potentials like other invertebrates and vertebrates, which suggests that circuit functions differ across different species. Only a few related studies have been performed in primates, which could be due to technical risk (Joshi and Hawken, 2006; Mitchell et al., 2007).

In this review, we first summarized most recent applications of the *in vivo* patch-clamp recording technique in the study of neuroscience. Then, we discussed its unique advantages and its possible combination with other techniques, such as two-photon imaging and optogenetics. Finally, some of the key factors of a successful *in vivo* patch-clamp experiment and possible solutions based on previous reports and our personal experience were discussed together.

## General Description of *In Vivo* Patch-Clamp Technique

**Figure 1:**
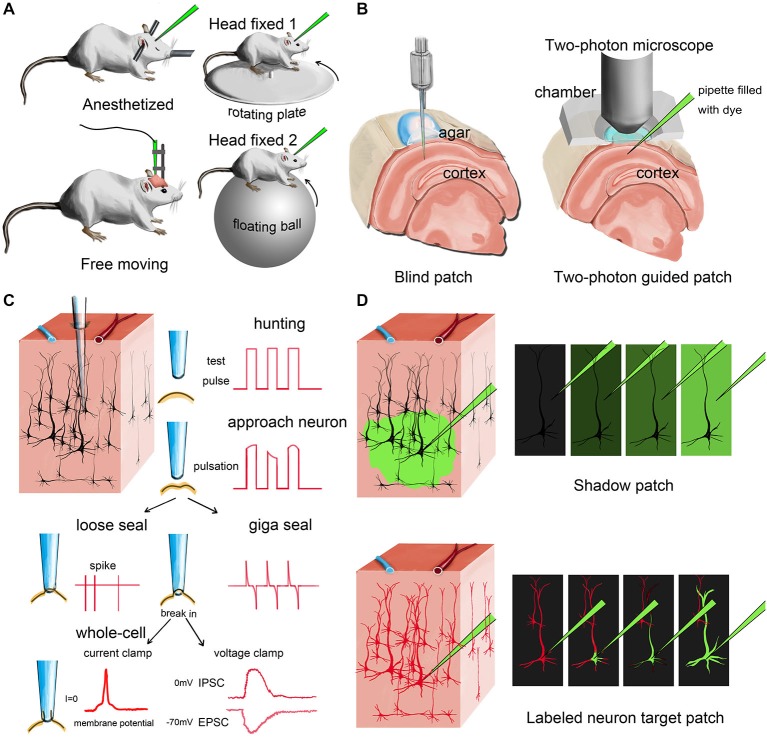
**Different modes of *in vivo* patch-clamp recording. (A)** Representative *in vivo* patch-clamp setups for anesthetized, awaking and behaving animals. **(B)** Demonstration of blind patch and two-photon-guided patch. **(C)** Procedures and different recording modes of *in vivo* patch clamp (blind patch). When the pipette approaches a nearby cell, heartbeat-associated changes become notable in test pulses. Releasing positive pressure allows the pipette tip to form a loose seal or a giga seal for loose-patch recording or whole-cell recording, respectively. After giga seal formation, the cell membrane can then be broken for either current-clamp recording or voltage-clamp recording. **(D)** Two different methods for visually guided *in vivo* patch clamp: shadow patch and labeled-neuron-guided patch.

*In vivo* patch-clamp recording can be performed in both anesthetized and awake animals. In the anesthetized state, the animal’s heart rate and breathing is relatively stable and smooth. This helps to minimize pulsation and increases the system’s stability, which is critical for any *in vivo* recording. Meanwhile, many higher brain functions, such as cognition, can only be studied in animals that are awake or even free moving. Whether anesthesia should be performed is largely dependent on the scientific questions raised and the design of the experiment (Figure 1A).

After anesthesia, fixation and surgery, the recording pipette is moved to the target region under a stereoscope, penetrating the pia matter. The cell-hunting stage is next. Based on whether the hunting procedure is visually guided or not, the cell-hunting stage can be classified into two approaches: blind or visually guided (Figure 1B).

### Blind Patch (Figure 1C)

Margrie et al. firstly systematically introduced the *in vivo* blind-patch procedure in 2002 (Margrie et al., 2002). In blind-patch mode, the recording pipette is moved forward to hunt for cells without visual guidance. Electrophysiological signals read from the pipette tip can provide helpful information. A change in seal resistance reflects the distance between the pipette tip and nearby neurons. An increase in pipette resistance and the occurrence of tiny spikes and pulsation-like waveforms may indicate that the pipette is approaching a nearby cell. The recorded spike shapes can provide helpful clues to identify the cell type of the recorded neuron (presumably). For example, excitatory pyramidal cells usually have a longer trough-to-peak interval than parvalbumin-expressing (PV+) inhibitory interneurons (Zhou et al., 2010; Moore and Wehr, 2013; Li et al., 2014a). To further verify the type and morphological details of the recorded neuron, fluorescent dye or biocytin can be added to the internal solution in the recording pipette, then researchers can reconstruct cell morphology after recording (Joshi and Hawken, 2006; Suzuki and Bekkers, 2010; Šišková et al., 2014).

A major advantage of blind patch is that the whole setup is much less complex compared to the visually guided configuration because no imaging device is needed (Figure 1B). Second, this simplicity provides more flexibility (e.g., space and penetrating angle) for researchers to combine *in vivo* patch-clamp with other techniques. Third, the recording depth is not limited by the imaging technique. For visually guided methods, the possible depth of imaging is generally less than 500 μm for most two-photon imaging setups, although imaging tissue at a depth of more than 1 mm has also been reported (Theer et al., 2003; Kobat et al., 2011).

### Visually Guided Patch (Figure 1D)

**Figure 2:**
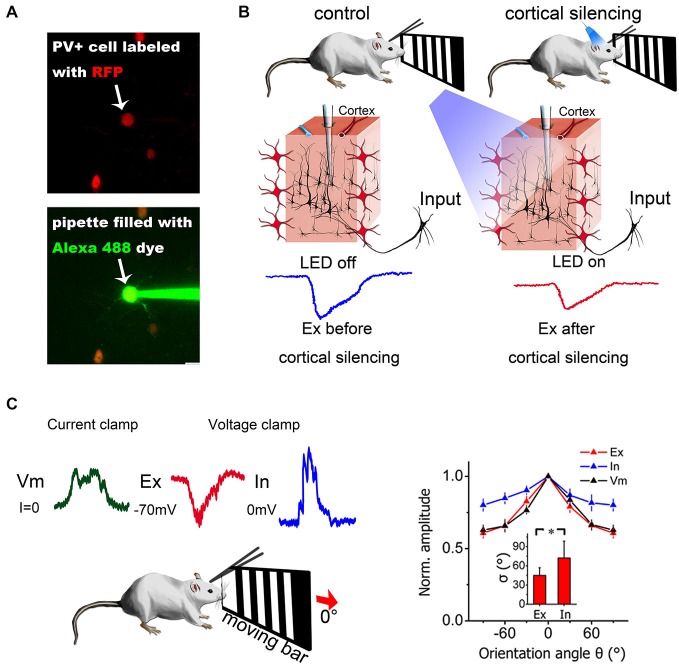
**Combination of *in vivo* patch-clamp recording and other techniques. (A)** Two-photon guided patch clamp can target specific neurons using fluorescent guidance. RFP, red fluorescent protein. Alexa 488, a green fluorescent dye widely used for two-photon imaging. **(B)** Optogenetic manipulation of neural circuits can isolate different sources of excitation. Blue light can activate ChR2-expressing PV+ inhibitory neurons in the visual cortex (red neurons) and silence cortical excitatory neurons. Then, the excitatory contribution from the thalamus only (thalamic EPSCs) can be isolated from the mixed input (thalamic + intracortical). Modified from Lien and Scanziani (2013), with permission. **(C)** Current-clamp and voltage-clamp recordings made from the same neuron in a living mouse. Current-clamp mode can record membrane potential changes and voltage-clamp mode can dissect synaptic currents into excitatory and inhibitory components by holding the cell membrane potential at different levels. The synaptic contributions to orientation selectivity can then be compared and quantified. The right panel depicts the orientation tuning curve for excitatory input (Ex), inhibitory input (In) and membrane potential (Vm). Error bar = SEM. The tuning width (delta) is denoted in the inset. *p < 0.01, paired *t*-test. Modified from Liu et al. (2011), with permission.

Compared with the blind-patch procedure, cell location and morphology can be visualized during the recording session in visually guided patch-clamp, which is very useful for recording from specific neurons (e.g., sparsely distributed inhibitory interneurons). To visualize a target neuron *in vivo*, the cell needs to be either brightened or shadowed. The brightening method uses transgenic or viral methods to visualize the neurons by adding fluorescent protein to the cell membrane (Trachtenberg et al., 2002; Komai et al., 2006; Häusser and Margrie, 2014; Li et al., 2014a). Then, a pipette filled with fluorescent dye can be used guide the hunt for the target neuron (Figure 2A). In the shadowing method, the extracellular matrix surrounding the target region is perfused with a fluorescent dye and brightened; thus, target neurons can be visualized by negative signals (Kitamura et al., 2008).

Due to the limitations of current imaging methods, the visualization quality at deep depths (>500 μm) is usually poor. To visualize deep nuclei, several alternative methods have been adopted. For example, Grienberger et al. removed the superficial cortex to expose and visualize hippocampal neurons directly (Grienberger et al., 2014; Velasco and Levene, 2014). Horton et al. used three-photon microscopy to see deeper (Horton et al., 2013). Because the working distance of the two-photon imaging technique is small, the imaging microscope might present a major limitation of the possible angle for pipette penetration, thereby increasing the difficulty of operation. Moreover, the high cost of two-photon imaging setups limits its popularity, especially for smaller labs without imaging facility support.

After cell hunting, the cell membrane can be kept intact for loose-patch recording or broken for whole-cell recording, which is very similar to traditional patch-clamp recording. Generally, the technical difficulty increases from *in vivo* loose-patch recording to *in vivo* whole-cell recording, but either mode can provide different types of valuable neuronal information.

## Unique Advantages of *In Vivo* Patch-Clamp

### “Input” and “Output” can be Retrieved from the Same Neuron

Loose patch means the pipette tip and the cell membrane are relatively close but not giga-sealed. In this mode, the pipette can record extracellular activity from only one nearby neuron, and the cell membrane can remain intact during recording. Compared with other extracellular recording methods, loose patch can record the spiking activity of a single neuron with a high signal-to-noise ratio. Typically, the ratio between the amplitude of the spike and baseline would be no less than 20. Moreover, both the local field potentials (LFPs) and single neuron spiking can be obtained simultaneously. The recording duration can also meet the needs of most *in vivo* experiments. In optimal conditions, loose-patch recording from a single cell can last for hours, even in awake animals (Joshi and Hawken, 2006; Runyan et al., 2010; Li et al., 2012; Bengtsson et al., 2013). A change in spike shape during the recording might indicate that the cell membrane is being gradually torn by repetitive rubbing from the pipette tip due to animal pulsation.

For whole-cell recording, a tight seal between the pipette tip and the cell membrane first needs to form (giga Ohm seal resistance, also known as “giga seal”), and then the cell membrane must be broken to access intracellular dynamics. Depending on whether the membrane current or potential is being manipulated, the recording can be divided into current- and voltage-clamp mode. Under current-clamp mode, researchers can inject current into the cell and monitor the change in membrane potential. However, for most *in vivo* current-clamp applications, no inward or outward current is injected (Liu et al., 2007, 2011; Jia et al., 2011). Under these conditions, current-clamp recording is similar to traditional intracellular techniques using sharp glass pipettes. Moreover, researchers can also use the current-clamp recording mode to monitor the condition of the recorded neuron and identify its cell type (e.g., regular-spiking or fast-spiking) based on the pattern of neuronal responses, which is similar to *in vitro* studies (Butt et al., 2005).

Compared with loose-patch recording, current-clamp recording can be used to monitor both sub- and supra-threshold potential changes simultaneously. Sub-threshold responses evoked by a certain stimulus reflect the synaptic input received by the recorded neuron, and supra-threshold responses represent the spiking output generated by the neuron (Wang et al., 2007). It has also been shown that in layer 4 pyramidal cells of the primary auditory cortex, the tuning of the onset latency of synaptic input is weaker than that of spiking output, which suggests that intracortical integration might contribute to enhancing tuning properties (Zhou et al., 2012b).

### Synaptic Contributions can be Dissected by Whole-Cell Voltage-Clamp Recording

The interplay between excitatory and inhibitory neurons provides the foundation for various functions in neuronal networks. For example, in sensory cortex, synaptic excitation and inhibition can control gain and modulate feature selectivity (Li et al., 2013). It has also been shown that excitation and inhibition wax and wane during spontaneous cortical oscillations (Sun and Dan, 2009). Understanding the spatial and temporal relationships between the two components can substantially facilitate identification of the mechanisms of neuronal functions. Using *in vivo* voltage-clamp recording, researchers can separate synaptic excitation and inhibition directly in real time (Figure 2C) by holding the membrane potential at −70 mV, which is the reversal potential of inhibitory currents (Cl^−^ ion channels), and at 0 mV, which is the reversal potential of excitatory currents (Na^+^ and K^+^ ion channels) (Zhang et al., 2003; Wu et al., 2006). A recent study also confirmed that the actual reversal potential is quite close to the theoretical value calculated from the Nernst equation (Ono and Oliver, 2014). By comparing synaptic input and spike output, researchers can easily determine whether certain properties or functions are purely inherited from presynaptic neurons or are generated *de novo*. In addition, there are also modeling-based methods that can be used to dissect excitation and inhibition. For example, Priebe et al. measured the membrane potential of a recorded neuron while injecting different currents and extracted the excitatory and inhibitory conductance by modeling (Priebe and Ferster, 2005). Similar estimations could also be obtained by holding the membrane potential at different hyperpolarization levels (not −70 mV and 0 mV as mentioned above) using voltage-clamp recording (Wehr and Zador, 2003).

*In vivo* voltage-clamp recording was first used in the 1990s to study the conductance changes evoked by visual stimuli in cat visual cortex (Pei et al., 1991; Borg-Graham et al., 1996) and was later applied to other neural systems, such as the auditory and somatosensory cortices. In the visual system, basic properties, such as receptive fields and orientation and direction selectivity, have been well studied using *in vivo* voltage-clamp recording techniques (Liu et al., 2011; Zhang et al., 2011; Bortone et al., 2014). In the auditory system, especially the primary auditory cortex, *in vivo* voltage-clamp recording has helped to reveal excitatory-inhibitory interactions and their role in many important auditory functions (Wehr and Zador, 2003; Scholl et al., 2010; Zhou et al., 2014). In the somatosensory cortex, functional projections (Kinnischtzke et al., 2014) and development (Minlebaev et al., 2011) have also been studied using *in vivo* voltage-clamp recording.

To obtain pure inhibitory synaptic input, the membrane potential of the recorded neuron needs to be held at 0 mV, which will inevitably activate voltage-gated channels and distort the measured conductance. So fast Na+ channel antagonist, especially QX-314, is usually added in the pipette solution to suppress spikes to record pure inhibitory synaptic input, which is not needed in current-clamp recordings (Fortune and Rose, 2003; Scholl et al., 2010; Sun et al., 2010; Adesnik et al., 2012). The internal solution is usually cesium-based for voltage-clamp recording, compared to the potassium-based solution used for current-clamp recording, for the same reason (Poo and Isaacson, 2011; Li et al., 2012). Meanwhile, there have also been reports that potassium-based internal solution can be used to hold the membrane potential at 0 mV without the aid of QX-314 (Poo and Isaacson, 2011) or Cs+ (Zhou et al., 2012a).

As mentioned above, voltage-clamp recording requires a high-quality cell membrane break-in, which provides a low series resistance and permits a valid command holding potential (Wehr and Zador, 2003). The series resistance in most *in vivo* patch-clamp experiments is typically within the range of 20 MOhms and 50 MOhms (Wehr and Zador, 2005). A higher series resistance could increase the difference between the actual holding potential and the command potential and lead to inaccurate reading of synaptic inputs. During the whole recording session, the series resistance needs to be monitored frequently to ensure the quality and reliability of recording. Meanwhile, other factors, such as the cable effect and space-clamping errors, should also be taken into consideration (Johnston and Brown, 1983; Spruston et al., 1993).

## Combination with Other Techniques for Better Functional Dissection

### Two-Photon Imaging

A patch-clamp pipette can be visually guided to target a specific neuron under two-photon imaging guidance. With two-photon imaging and transgenic animals, it is possible to visualize a PV+ neuron alone for precise patch-clamp recording (Figure 2A). This can largely simplify the procedures (e.g., immunohistology) required to identify the type of neuron being recorded. Many recent studies have tried to clarify the distinct yet interesting features of different interneurons in the cortex by using a combination of these methods (Runyan et al., 2010; Ebina et al., 2014; Li et al., 2014a).

### Optogenetics

By combining optogenetics and *in vivo* patch-clamp recording, it is possible to directly quantify the synaptic contributions of certain sources and to obtain even better functional dissection of neural circuits. Two research groups (Li et al., 2013; Lien and Scanziani, 2013) have used this method to isolate the thalamocortical contribution from the total excitatory input and revealed the function of cortical amplification (Figure 2B). In the auditory cortex, different research groups have found partially controversial tuning properties in inhibitory neurons when both *in vivo* whole-cell patch-clamp recording and optogenetics are used (Moore and Wehr, 2013; Li et al., 2014a).

## Key Factors of a Successful *In Vivo* Patch-Clamp Experiment

### Success Rate

For whole-cell recording, Margrie et al. reported their success rate as close to 20% of penetrations under optimal conditions (Margrie et al., 2002). Zhou et al. also reported that they could obtain one good whole-cell recording in one head-fixed awake animal on average (Zhou et al., 2014). As mentioned above, the formation of a giga seal is a critical step for whole-cell recording. It is known that a clean pipette tip is critically important to obtain a successful giga seal (Hamill et al., 1981), and proper positive pressure during penetration is helpful in keeping the pipette tip and cell membrane clean. Successful membrane break-in could result in a low series resistance, which determines the holding quality. Margrie et al. reported that a slow ramp of negative pressure (20–250 mbar) has a higher success rate than rapid suction for membrane break in. However, this operation is still highly dependent on the experience of the experimenter and the type of recorded neuron. Moreover, success rates significantly decrease with increasing animal age and recording depth, which is similar to previous findings in slice recording (Margrie et al., 2002).

Moreover, because the recording is performed in living animals, it is easier for membrane debris to block the pipette tip or “repair” the broken membrane (also known as “reseal”) during a whole-cell recording due to pulsation. This could also limit the recording duration of a successful *in vivo* patch-clamp experiment. A well-prepared experiment can make the best use of the valuable recording time.

### Recording Depth

In most cases, *in vivo* patch-clamp recordings are performed in superficial regions. *In vivo* whole-cell recording from neurons 2~5 mm below the brain surface, such as in the hippocampus (Harvey et al., 2009) or thalamus (Margrie et al., 2002), has also been reported. There is no clear limitation of recording depth for *in vivo* patch-clamp recording. But the pipette tip is more easily contaminated when penetrating into deeper nuclei. One possible solution is to use a “guiding tube” to create a clean path for the recording pipette (Kuo and Wu, 2012). There are also other methods, such as the removal of superficial tissue to expose the recording area (Zhou et al., 2012a). It remains more difficult to perform patch-clamp in deeper nuclei such as the basal ganglia *in vivo*, so experimenters have chosen different ways, such as ex-vivo patch-clamp (Brigman et al., 2013). For visually guided patch-clamp, the recording depth is also limited by the imaging microscope. So far, most two-photon imaging systems can only reach ~700 μm below the surface. However, the signal-to-background ratio decreases with imaging depth, which makes patching deeper cells using visual guidance more difficult (Horton et al., 2013).

## Discussion

The major limitation of *in vivo* patch-clamp recording is its technical difficulty, which requires experienced personnel and a large amount of patience. A training period of 3–6 months is reasonable for a graduate student/research assistant without prior experience. Kodandaramaiah et al have developed a robot to do some of the laborious procedures (Kodandaramaiah et al., 2012). This setup can automatically insert the pipette into the brain tissue via a linear actuator controlled by computer program. Meanwhile, the seal resistance is monitored and used to judge whether the pipette has reached a cell or not. Then, negative pressure is applied to form a giga seal and suction or a “zap” voltage pulse can be applied to break the membrane. All of these pressure-switch operations are controlled by a set of programmable valves. Although this solution is only partly automated (you must change the pipette manually), a completely automated *in vivo* patch-clamp setup is expected to be available in the near future as more research groups become interested in applying this tool to facilitate their research work.

Like any technique or method used for scientific research, *in vivo* patch-clamp recording is imperfect. There are arguments debating whether it can really reflect the properties of synaptic inputs received by the recorded neuron, or it is just a measurement of synaptic currents within a limited range near the recording site. Also, in blind-patch recording mode, extra experience is needed to tell the difference between spikes recorded from the soma or from dendrites. Nevertheless, *in vivo* patch-clamp recording is still the best choice to quantify synaptic contributions in living animals. It has been attracting more attention in the neuroscience field, as demonstrated by the increasing number of high-quality research papers published in recent years. Particularly for researchers who are interested in intact neural circuits, *in vivo* patch recording could be very helpful if properly combined with other tools, such as optogenetics and two-photon imaging.

## Conflict of Interest Statement

The authors declare that the research was conducted in the absence of any commercial or financial relationships that could be construed as a potential conflict of interest.
